# Giardiasis Pattern among Different Age Categories: Childhood Assemblage B Proclaim Endemicity

**Published:** 2019

**Authors:** Doaa A. AHMED, Mona A. RABBO, Manal JAMJOOM, Hala S.SALEM, Marwa A. GHIETH

**Affiliations:** 1. Department of Parasitology, Faculty of Medicine for Girls, Al-Azhar University, Cairo, Egypt; 2. Department of Medical Microbiology and Parasitology, Faculty of Medicine, King Abdul Aziz University, Riyadh, Saudi Arabia; 3. Department of Medical Parasitology, Faculty of Medicine, Beni Suef University, Beni-Suef, Egypt

**Keywords:** Giardiasis, Assemblage, Genotyping, PCR-RFLP, *gdh*, *bg*, *tpi*

## Abstract

**Background:**

Giardiasis is one of the commonest intestinal parasitic diseases that affects wide range of age groups. We aimed to detect the pattern of *Giardia intestinalis* assemblages among symptomatic patients at the age of 2 up to 40 years.

**Methods:**

Stool samples were collected from 278 patients and examined microscopically and genetically for giardiasis. *Giardia* was diagnosed using wet mount examination and subjected to molecular assays targeting three genes, glutamate dehydrogenase (*gdh*) using semi-nested PCR (nPCR), β-giardin (*bg*) and triose phosphate isomerase (*tpi*) using nPCR. Amplified products were subjected to genotyping using PCR-restriction fragment length polymorphism (PCR-RFLP) targeting *gdh* and *bg* genes.

**Results:**

Among 48 samples positive by microscopy and by a minimum of one of the three used genes, genotyping was successful among 23 samples (47.9%). Assemblage B was more prevalent (16/23, 69.6%), than assemblage A (4/23, 17.4%) and 3 (13%) isolates were identified as assemblage B at *gdh* locus which later were identified as assemblage A at *bg* locus. Sub-assemblage AII (3/4, 75%) and sub-assemblage BIII (12/15, 66.7%) were predominate at *gdh* locus. Age groups was an estimated risk factor for infection with assemblage B with a peak (87.5%) during 6 to 12 years (*P*< 0.05), diarrhea and abdominal pain (OR (95%CI) = .654 (.094, .963); .201 (.048, 1.009), respectively) were significantly associated with assemblage B.

**Conclusion:**

It is recommended to suspect infection with giardiasis assemblage B by physicians during late childhood presenting with diarrhea and abdominal pain.

## Introduction

Giardiasis is a feco-oral transmitted protozoan infections that manifested by variations of clinical symptoms (1, 2). The disease is asymptomatic, self-limiting among patients in chronic area and immuno-competent (3). Severe form of diarrhea, malabsorption and even weight loss may develop. In between, variety of symptoms such as steatorrhea, abdominal pain, flatulence and nausea are present (4, 5). *Giardia* can affects human from childhood till seniors depending on immunity state (6).

Host related factors, genetic diversity and virulence of the parasite play a role in clinical presentation of the disease (7).

Genetically, *Giardia* have eight assemblages (A-H) that are host specific, however both assemblage A and B are commonly detected among humans (8, 9). *Giardia* Genotyping is helpful in demonstration of source of infection and detect if there is a zoonotic potential of the disease (10).

To clarify the prevalence of giardiasis among symptomatic patients with different age groups and figure out if a probable age related genetic heterogeneity, PCR-RFLP genotyping addressing *gdh* and *bg* was used for genotyping of *Giardia* and supposing risks and associated factors of assemblage patterns by this study.

## Materials and Methods

### Study design and populations

In this cross sectional study, we included 278 symptomatic patients with age range from 2 to 40 years attending the Outpatients clinics at Al-Zahraa Hospital, AL-Azhar University, Egypt. Patients suffering from diarrhea, abdominal pain, vomiting and abdominal distension were included. Samples were taken from patients after informing them about the aim of the study and they were free to refuse samples collections. A questionnaire including demographic and clinical data was filled by all patients or their parents.

### Samples collection and processing

A minimum of one faecal sample was collected from all cases. Samples were divided into 2 parts, one for coproscopic analysis by direct wet mount using Lugol's iodine and saline, the second part was kept frozen for further techniques. Multiple smears from each sample were examined before formal ethylacetate concentration technique and after to detect *G. lamblia* cyst and/or trophozoites.

Informed consent was obtained from patients and parents of children included in the study and they were informed about purpose of the study. The protocol was approved by Al-Azhar University Research Ethics Committee. Sample collections and ethics were in agreement with the Helsinki declaration, 1964.

### Copro-molecular assays

Genomic DNA extraction for all microscopically positive samples for *Giardia* was done using QiAmp® stool DNA extraction kit (QIAGEN, Hilden, Germany) according to the manufacturer’s protocol. With a modification in the form of allowing samples to be vortexed for 10 minutes after mixing with glass peads before processing of DNA Extraction. Eluted DNA samples were kept at –4 ºC for further processing.

### PCR amplification

Genetic identification of *Giardia* isolates was done targeting three genes *gdh, bg* and *tpi*. Using semi nPCR for *gdh* and nPCR for *bg* and *tpi*. Genotyping was done using PCR-RFLP targeting *gdh* and *bg.*

### gdh

Semi-nested PCR (nPCR) was used for amplification of ∼432 bp fragment for *gdh* locus through using the following primers, GDHeF: 5′ TCAACGTYAAYCGYGGYTTCCGT 3′ for primary PCR reaction, GDHiR: 5′ GTTRTCCTTGCACATCTCC 3′, and GDHiF: 5′ CAGTACAACTCYGCTCTCGG 3′, for secondary PCR. The reaction mixture was up to 25 μl and conditions were done according to Read et al. (11). The amplified products were stained by ethidium bromide then visualized with 1.5% agarose gel electrophoresis.

### PCR-RFLP of the gdh

The PCR amplified products were subjected to digestion by two restriction enzymes *NIa IV* (New England Biolabs. 0141210) and *RSaI* (New England Biolabs. FD1124) for assemblages and subassemblages discrimination according to the manufacturer’s protocol. Then restricted profiles were visualized with 2 % agarose gel electrophoresis after ethidium bromide staining.

### bg

The reaction conditions and primers were done according to Caccio et al. (12), amplification of *bg* gene fragment of nested PCR through using: External forward primer G7: AAGCCCGACGACCTCACCCGCAGTGC and reverse primer G759: GAGGCCGCCCTGGATCTTCGAGACGAC, amplifying a DNA fragment of ∼753 bp. Then second reaction was done using the Internal Forward Primer BGF: GAACGAACGAGATCGAGGTCCG and Internal Reverse Primer BGR: CTGGACGAGCTTCGTGTT, for amplification of ∼511 bp.

### PCR-RFLP for bg

The amplified PCR products were subjected to digestion enzymes using the restriction endonucleases *Hae III* to distinguish assemblages A, B, C, D, E and F. 2% agarose gel electrophoresis stained with ethidium bromide was used for visualization of the reactions profiles.

### tpi

Nested PCR (n PCR) reaction conditions and primers were performed according to Sulaiman et al. (13), using two sets of primers, AL3543: 5′- AAATIATGCCTGCTCGTCG-′ 3 and AL3546: 5′- CAAACCTTITCCGCAAACC-′ 3 for the first reaction, AL3544: 5′- CCCTTCATCGGIGGTAACTT-′ 3 and AL3545: 5′- GTGGCCACCACICCCGTGCC-′ 3 for the second reaction. Amplifying DNA fragment of ∼605 bp at the primary reaction and ∼530 bp at the secondary reaction.

### Statistical analysis

For data analysis, the statistical package of social sciences (SPSS) version 20 was used. Descriptive data were analyzed by mean ± standard deviation, while qualitative data were analyzed by frequencies. Comparing data was done using Chi-Square, student T test, and univariate logistic regression test calculating the 95% confidence interval (CI) and odds ratio (OR) to estimate the sociodemographic risk factors and associated clinical data with each assemblage.

## Results

### Giardiasis detection by microscopy and PCR

**Fig. 1:**
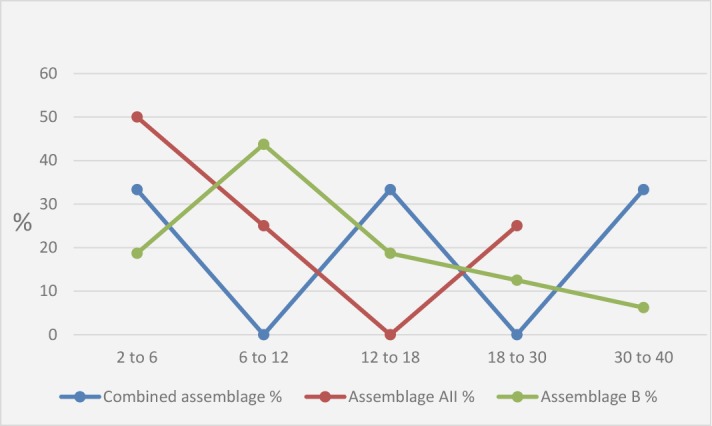
*Giardia* assemblage distribution among different age groups

Out of 278 symptomatic patients with age range from 2 to 40 years old with a mean age of 9.7±8.67, 55.1% male and 44.9% female, microscopic examination revealed *Giardia* cyst or/and trophozoites in 48 cases, and proved by detection of PCR products for *Giardia* at the expected size by at least one of the three used genes (*gdh, bg* and *tpi*). *tpi* gene amplified product were not subjected to further processing, PCR-RFLP was performed at *gdh* and *bg* loci. Genotyping showed 23 cases (47.9%) that were successfully digested by enzyme using PCR-RFLP. Assemblage B was detected in 16 cases (69.6%), assemblage A in 4 cases (17.4%) and 3 (13%) isolates were identified as assemblage B at *gdh* locus which later were identified as assemblage A at *bg* locus (Fig. 1, Table 1).

**Table 1:** Distribution of *Giardia* isolates among *gdh* and *bg* genes

***Variable***	***Assemblage pattern***			
***Giardia genes***	***A (n=4)***		***B (n=16)***	
	***no***	***%***	***no***	***%***
*gdh* (n=16)	3	75	12	75
*bg* (n=1)	0	0	1	6.25
*gdh* and *bg* (n=4)	1	25	3	18.75

### PCR-RFLP by gdh and bg

**Fig. 2:**
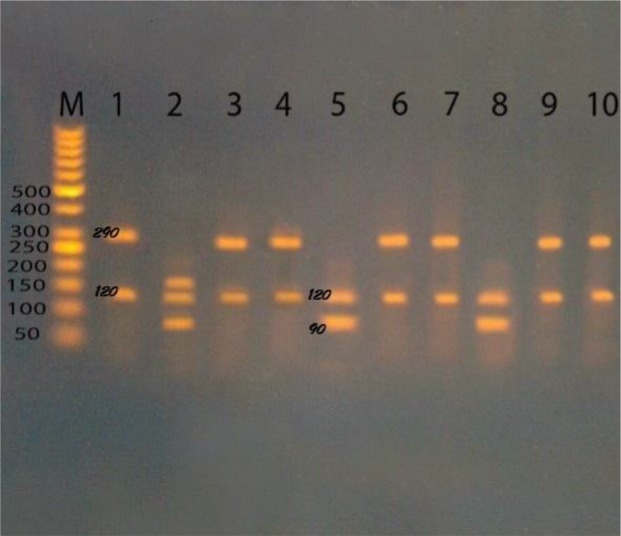
Agarose gel stained with ethidium bromide, M (50 bp ladder marker), lane 1,3,4,6,7,9,10 after digestion by *NIa IV* enzyme of PCR products targeting *gdh* gene as assemblage BIII,BIV at 290 and 120 bp. Lane 5,8 are assemblage AII at 90 and 120 bp, lane 1 is assemblage AI at 90,120 and 150 bp

Assemblage B was detected in 18 samples at *gdh* locus, 3 isolates of them were detected at both *gdh* and *bg* loci, subassemblage BIII, and mixed BIII, BIV (12, 4, respectively) were detected while 2 isolates of assemblage B failed to be subtyped. 3 isolates were subtyped as assemblage BIII at *gdh* locus then were identified at *bg* locus as assemblage A. Assemblage A was detected in 4 samples, 3 of that were subtyped as AII at *gdh* locus, and one sample was subtyped as assemblage AI (Fig. 2, Table 2).

**Table 2:** Assemblages and subassemblages of *Giardia* by *gdh* and *bg* genes

***no***	***gdh***	***bg***	***no***	***gdh***	***bg***
G1	B III	B	G13	BIII	
G2*	B III	A	G14	AI	
G3	B		G15	BIII	
G4	BIII,BIV	B	G16	BIII	
G5	BIII,BIV		G17	BIII	
G6	BIII	B	G18*	BIII	A
G7	BIII		G19	AII	A
G8	BIII,BIV		G20	BIII	
G9*	BIII	A	G21	BIII	
G10	B		G22	AII	
G11	BIII,BIV		G23	AII	
G12**	-	B			

* combined assemblages, isolates G2, G9, G18 were identified by *gdh* gene as assemblage BIII while by *bg* gene it was identified as assemblage A.

** isolates G12 was not digested by enzymes for *gdh* gene, however PCR product was detected by *gdh* using semi nPCR

**Fig. 3:**
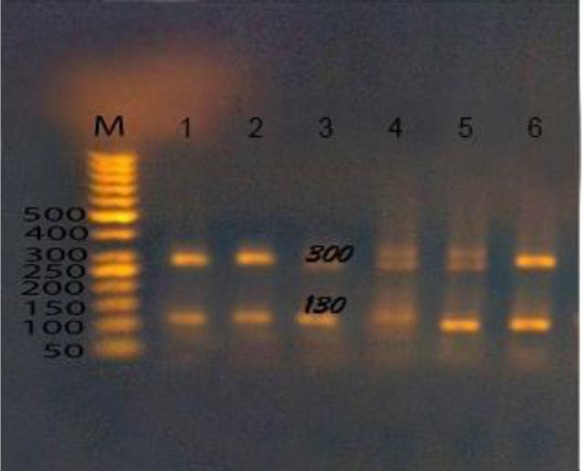
Agarose gel stained with ethidium bromide, M (50 bp ladder marker), lane 1,2,3,4,5,6 after digestion by *RSaI* enzyme of PCR products targeting *gdh* gene as assemblage BIII at 130 and 300 bp

Assemblage B was detected in 4 samples at *bg* locus, one isolates (G12) was only identified at *bg* locus as assemblage B and failed to be identified at *gdh* locus, while the other 3 samples were identified at both *gdh* and *bg* loci. Assemblage A was identified at 4 samples at *bg* locus, only one samples was identified by both *gdh* and *bg* loci while the other 3 samples were previously identified by *gdh* as subassemblage B (Fig. 3).

### Sociodemographic and clinical data in relation to assemblages

**Fig. 4:**
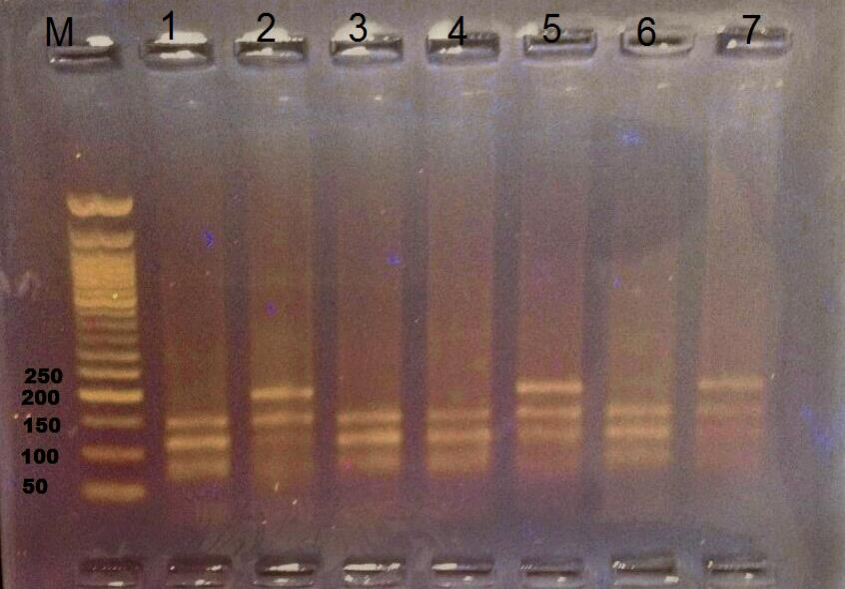
Agarose gel stained with ethidium bromide, M (50 bp ladder marker), assemblages pattern after digestion by enzymes *Hae III* enzyme of PCR product targeting *bg* gene, lane 1,3,4 ,6 as assemblage B at 150,117 bp lane 2,5,7 as assemblage A at 201, 150, 110 bp

Age group was an estimated risk factor for infection with assemblage B (*P* value < 0.05), assemblage B was presented among different age groups higher (7/8 children, 87.5%) in the age of 6–12, at the age 12–18 (3/4 children, 75%) and the age of 2–6 (3/5 children, 60%). Assemblage A was higher presented in the age of 2–6 (2/5 children, 40%). Male gender was common among both assemblage A and B (100, 62.5%, respectively). Statistical significant difference was found between assemblage B and diarrhea and abdominal pain (*P* value = 0.01, 0.02, respectively). None of sociodemographic data or clinical data was associated with assemblage A (Fig. 4, Table 3).

**Table 3:** Distribution of sociodemographic and clinical data among assemblages A and B

***Variable***	***Combined assemblage (n=3)***		**P *value***	***OR (95% CI)***	***Assemblage A (n=4)***		**P *value***	***OR (95% CI)***	***Assemblage B (n=16)***		**P *value***	***OR (95% CI)***
	***no***	***%***			***no***	***%***			***no***	***%***		
Age (yr)
2–6	1	33.3	.1		2	50	.6		3	18.7	.04*	
6–12			3		1	25			7			
12–18	0	0			0	0			3	43.7		
18–30		33.3			1	25			2			
30–40	101	033.3			-				1	18.712.56.2		
Gender												
Male	1	33.3	.4	.365	4	100	.4	2.15	10	62.5	.54	1.11
Female	2	66.7	0	(.013,1.21)	0	0		(.375,.418)	6	37.5		(.347,1,51)
Diarrhea												
Yes	3	100	.2	1.00	1	25	.17	.235	11	68.7	.01*	.654
No	0	0	0	(.89,1.13)		75		(.026,2.13)	5	31.3		(.094,.963)
Distension												
Yes	2	66.7	.6	1.21	2	50	.40	.389	9	56.3	.408	.724
No	1	33.3	8	(.102,1.14)	2	50		(.076,1.98)	7	43.7		(.220,1.659)
Abdominal pain												
Yes	1	33.3	.7	.906	1	25	.52	.693	13	81.3	.02*	.201
No	2	66.7	7	(.67,1.77)	3	75		(.119,4.02)	3	18.7		(.048,1.009)
Vomiting												
Yes	0	0	.7	1.01	1	25	.22	3.46	0	0	.360	.771
No	3	100		(.886,1.16)	3	75	7	(.504,1.137)	16	100		(.461,.748)

* significant *P* value (≤ .05)

## Discussion

In the present study, among gastrointestinal symptomatic patients, giardiasis was detected microscopically and molecularly targeting three genes *gdh, bg* and *tpi* among 48 (16.7%) cases. Genotyping using PCR-RFLP was successfully performed among 23 cases (47.9%) targeting *gdh* and *bg*. Assemblage B was more prevalent (69.6%) than assemblage A (17.4%). Isolates showing combined assemblages (13%) were identified at *gdh* locus as assemblage B and at *bg* as assemblage A.

Worldwide, assemblage B was dominating among symptomatic patients in England (14) and Spain (15). In addition to its predominance among different populations by many studies, at India (16), Bangladesh **(**17) and Kuala Lumpur (18).

On the contrary, assemblage A was more prevalent in Mexico (19) and Turkey (20). Impulsively, a study at Iran revealed a relatively equal pattern distribution of assemblage A and B (21).

Ecology of giardiasis is highly linked to the source of infection and whether a potential zoonotic of the disease occurs in some areas, it reflects the district human behavior explaining either an anthropogenic, zoonotic or mixed focus for disease transmission. In Egypt, many studies in last 5 years declared both assemblage A and B endemicity with predominance of genotype B (22, 23, 24). Assemblage B pattern is bound to human and bulk of animal infections, this echo the source of disease transmission to human activities and contamination of water sources.

However we cannot neglect a previous study **(**25) that reported dominant assemblage A and the remarkable results **(**26) reported assemblage E detection in Egypt. Distribution of assemblages and subassemblages pattern differs from area to anther even in the same country, it is multifactor relations to the source of infection, genetic diversity of the parasite and behavior of populations in each community for acquiring the disease.

In the current study, at *gdh* locus, 22 out of the 23 samples (95.6%) were successfully genotyped, 4 were typed as assemblage A and 18 were assemblage B that further subtyped as subassemblage BIII, and mixed BIII, BIV (12, 4, respectively), and 2 isolates failed to be sub-typed. At the *bg* locus, genotyping was successfully performed in 8 samples (34.7%). Assemblage B and A were detected in 4 samples for each (50%).

Performance of genotyping at *gdh* and *bg* was (95.6%, 34.7%, respectively). Similarly, Huey et al **(**27) reported *gdh* with higher number of amplicons detection than *bg* in contrast to Soliman et al. **(**28) reported 100% performance at *bg* locus compared to 46.6% at *gdh* locus.

Samples that failed to be detected at *bg* level may be attributed to the fact that they reside to other subtypes that don’t suit the used enzymes (29). Another technical cause that only related to our study conditions is that the amplified samples may be insufficient to be better digested by enzymes and unavailability of much DNA of the positive samples for re-amplifying PCR products targeting *bg* gene.

Our results showed predominance of subassemblage AII (75%) than subassemblage AI (25%), assigning that to the main source of infection which is anthropogenic among assemblage A populations of the present study.

The 3 isolates with combined assemblage pattern that were identified by *gdh* as subassemblage B and by *bg* as assemblage A, demonstrating that person infection with more than one assemblage, potential acquiring infection with a two genetically distinct parasitic cysts or reinfection of the previously infected host and presence of multisource for infection (30). A gene related cause is the conversely amplification of one locus over another locus by both *gdh* and *bg* (11).

As regards age groups, infection with assemblage B showed a peak in late childhood (6–12) (87.5%), followed by (75 %) the age of early childhood (2–6), with sustaining of infection among older age (30–40). Assemblage B showed command endemicity along childhood groups. Age group was an estimated risk factor for infection with assemblage B (*P* value < 0.05). While assemblage A showed a peak during younger age (2 to 6) of early childhood. No infection was reported above age of 30.

Similarly, El Basha et al **(**31) in Egypt mentioned assemblage B dominance among 6–16 age range. While assemblage A was common in 2–8 age range. Also, Lass et al **(**32) in Afghanistan reported assemblage B prevalence among school age children. Schoolchildren are more exposed to outdoor activities than younger ages, also they may neglect their hygiene or eating without washing hands making them more exposed to assemblage B infection that is prevalent among human and wide range of animals.

Assemblage pattern endemicity in a particular community draw the clinical outcome of giardiasis, thus introduction of genetically different subtype in an area may produce exaggerated symptoms and potentiate pathogenesis of infections (33).

In the present study, assemblage B was significantly associated with diarrhea and abdominal pain (*P* value = 0.01, 0.02, respectively). None of sociodemographic, gender or clinical data was associated with assemblage A.

Association of giardiasis symptomology and assemblage B was mentioned by many studies all over the world (7, 22, 34, 35). In contrast, others reported assemblage A clubbing with clinical symptoms over B (11, 36). Diversity among studies point to the virulence of parasite and immunity of host that differ from area to another.

## Conclusion

It is recommended to suspect infection with giardiasis assemblage B by physicians during late childhood presenting with diarrhea and abdominal pain.
